# Supplementary material for: Metal Accumulation Using a Bacterium (K-142) Identified from Environmental Microorganisms by the Screening of Au Nanoparticles Synthesis
Source: Materials (Basel). 2020 Nov 2;13(21):4922. doi: 10.3390/ma13214922 (PMC7662954; doi:10.3390/ma13214922)
Supplement: Supplementary file 1 [file materials-13-04922-s001.pdf]

materials

MDPI

Supplementary Materials

Metal Accumulation Using a Bacterium (K-142) Identified From Environmental Microorganisms by The Screening of Au Nanoparticles Synthesis.

Yiting Li and Michio Suzuki

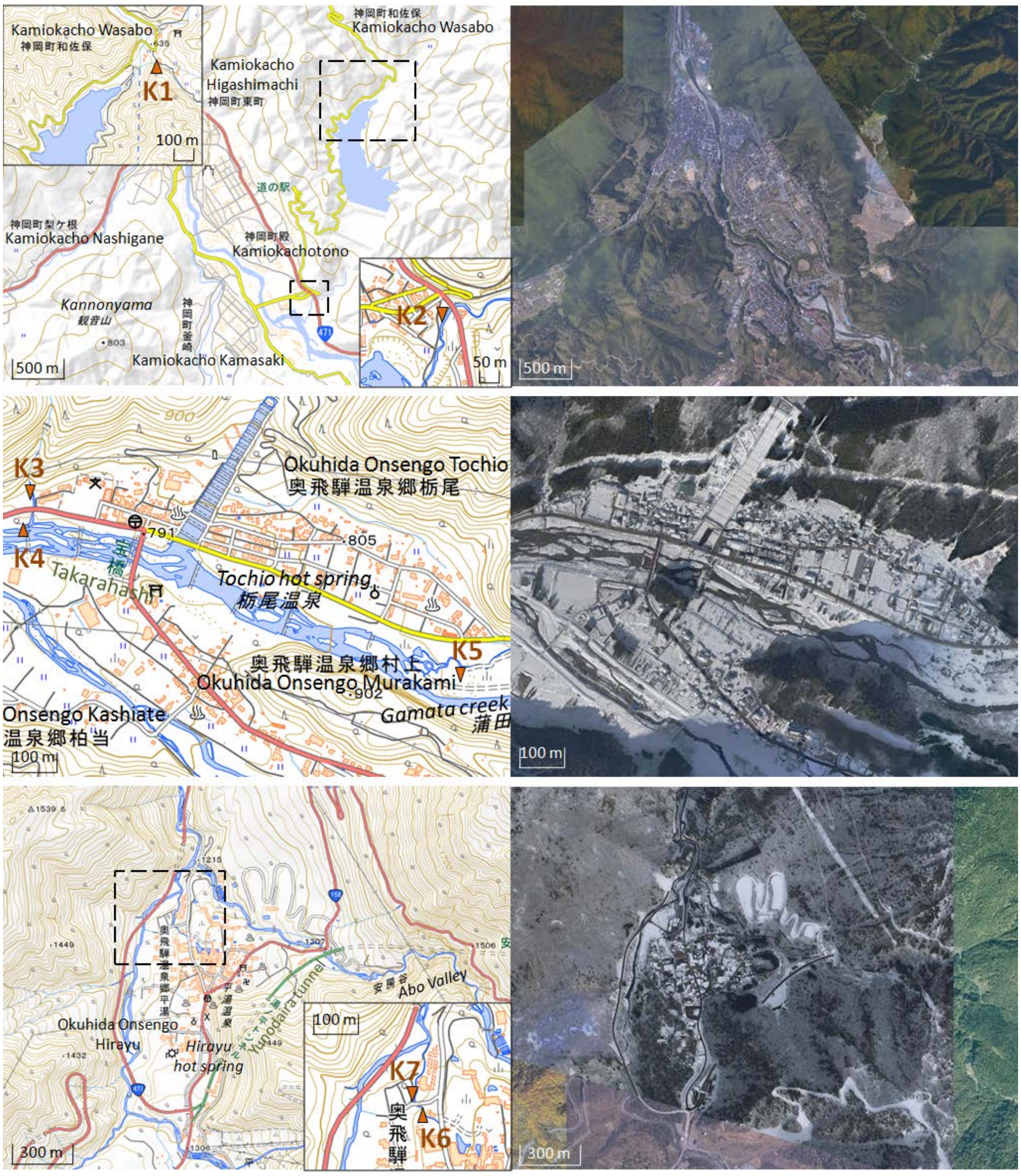

Figure S1. The spots where the river water was collected (around the Kamioka mine in Hida City, Gifu Prefecture in Japan). **K1**: 36.337625, 137.322322 (Kamioka-cho wasabo stream). **K2**: 36.319049, 137.313279 (the Takabara creek). **K3**: 36.248646, 137.512246 (the tributary of Gamata creek (near by the hot spring)). **K4**: 36.248282, 137.511903 (the tributary of Gamata creek (near by the bus stop)). **K5**: 36.244623, 137.525039 (the Gamata creek). **K6**: 36.194267, 137.550689 (the tributary of Takabara creek). **K7**: 36.194364, 137.550548 (the waterfall nearby trup farm). The map data quoted from GSI Maps.

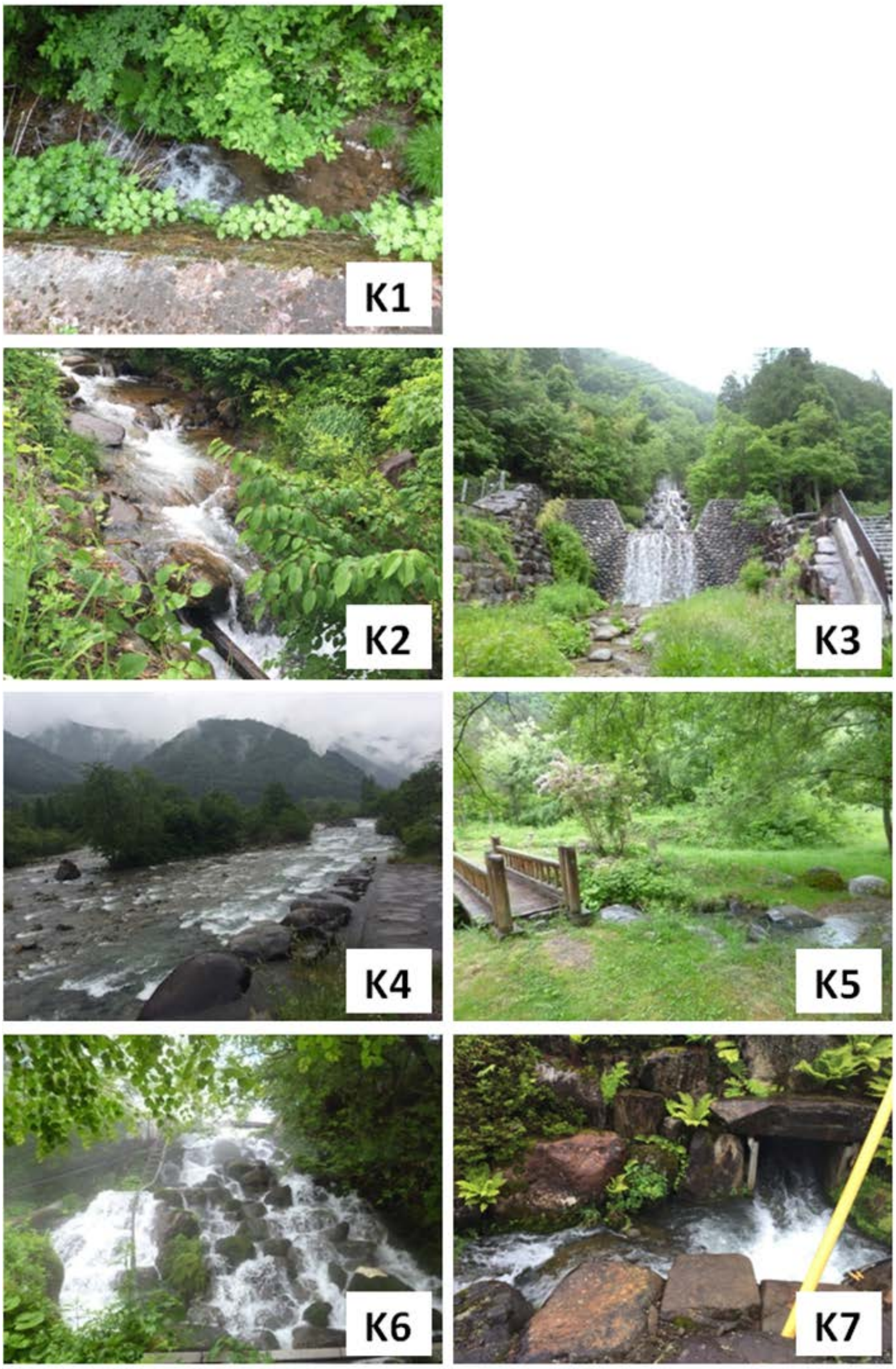

Figure S2. Photographs of the seven spots (K1-K7) where the environmental water was collected (around the Kamioka mine in Hida City, Gifu Prefecture in Japan).

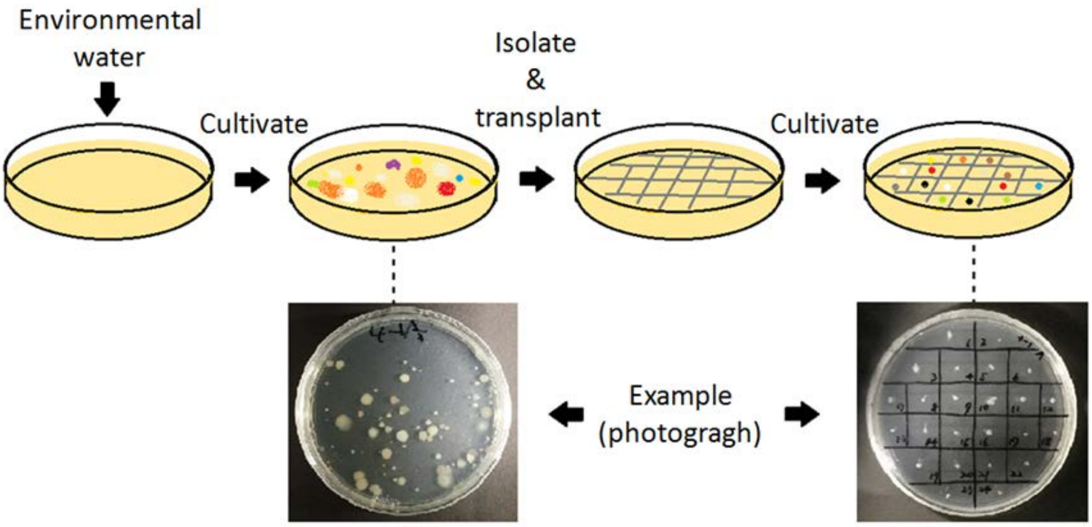

Figure S3. The process flow diagram of cultivation and isolation of the microorganisms in environmental water.

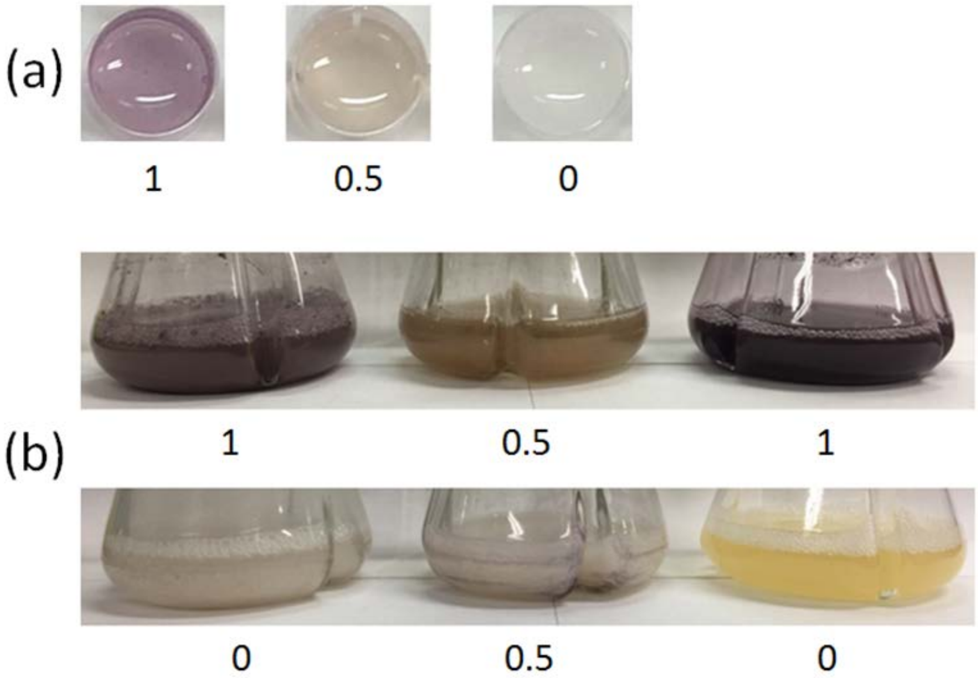

Figure S4. Examples of digitizing color changes. (**a**) Small amount synthesis. (**b**) Large amount synthesis.

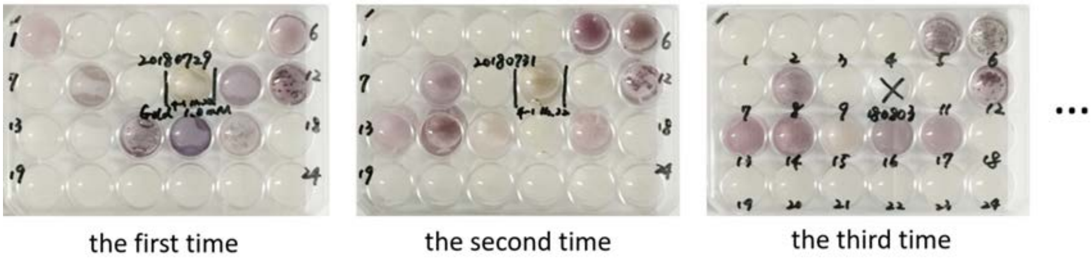

Figure S5. Example of reproduction experiments of Au nanoparticle synthesis (small amount).

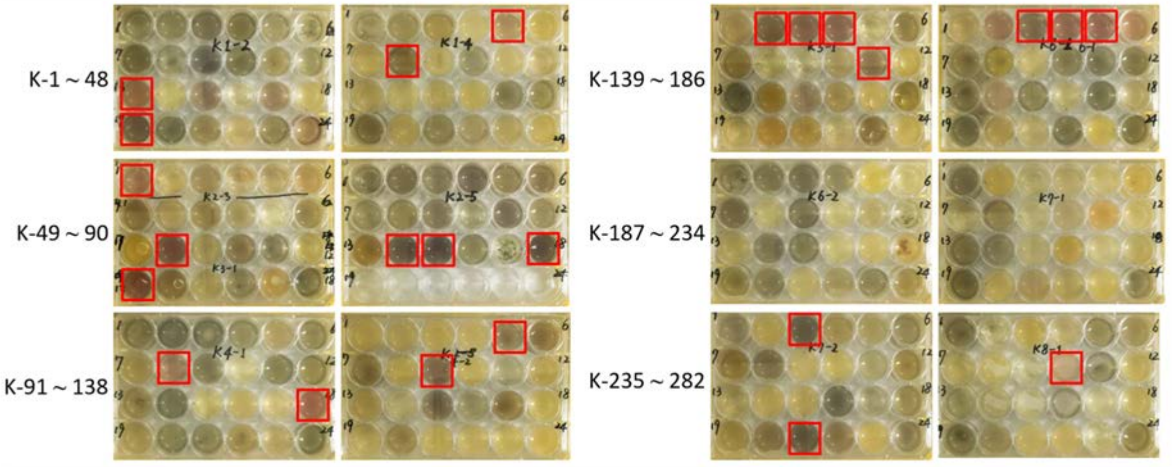

Figure S6. The color results of the first round screening. K-1~K-282 are the 282 target microorganisms isolated in this research. The red square frames indicate the 24 microorganisms selected in this round.

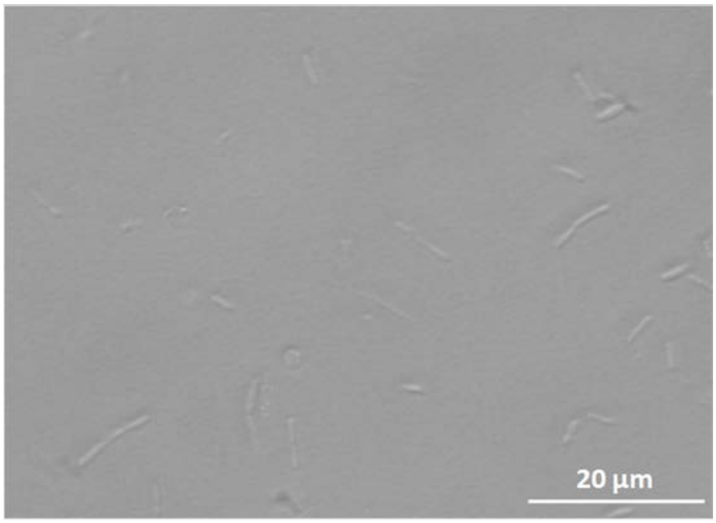

Figure S7. The optical microscope image of K-142.

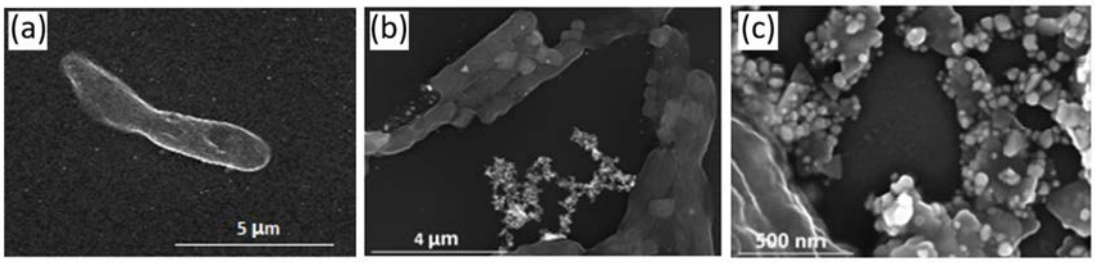

Figure S8. The SEM images of K-142. (**a**) Without auric acid (only cells). (**b**), (**c**) Cells added with auric acid.

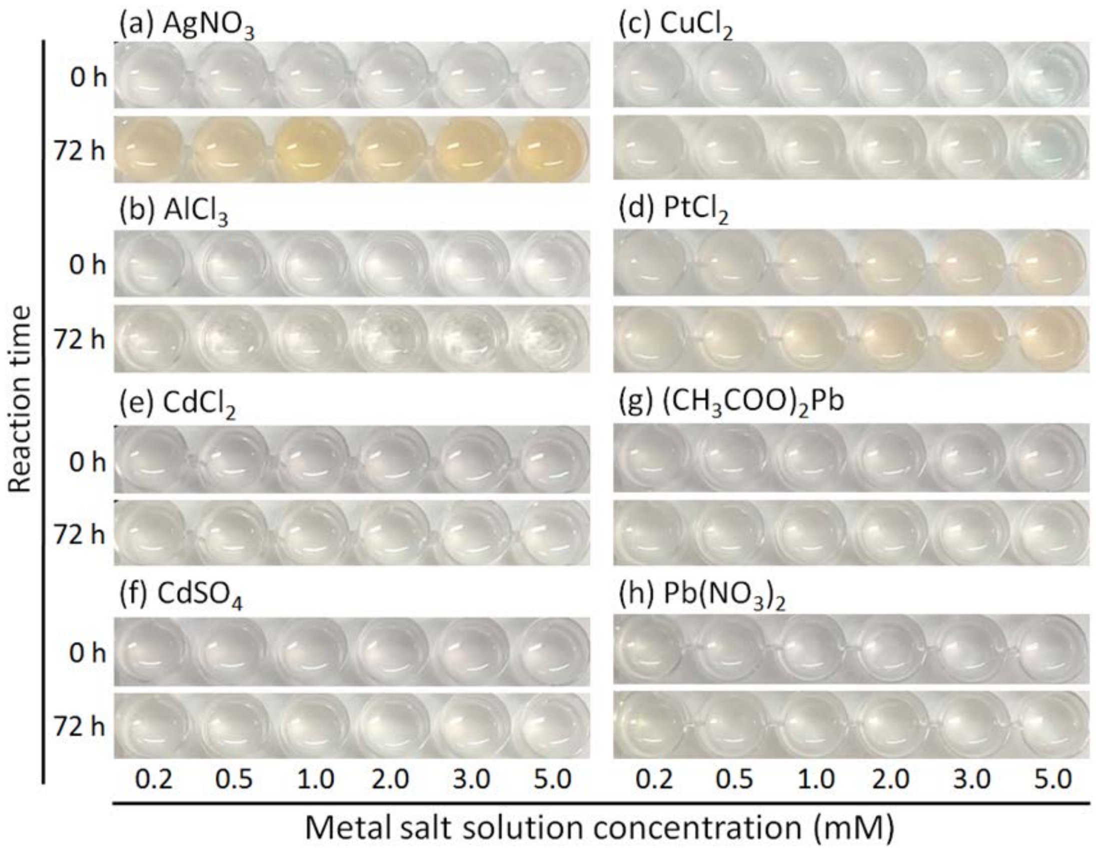

Figure S9. Color changes after the addition of metal salt solution.

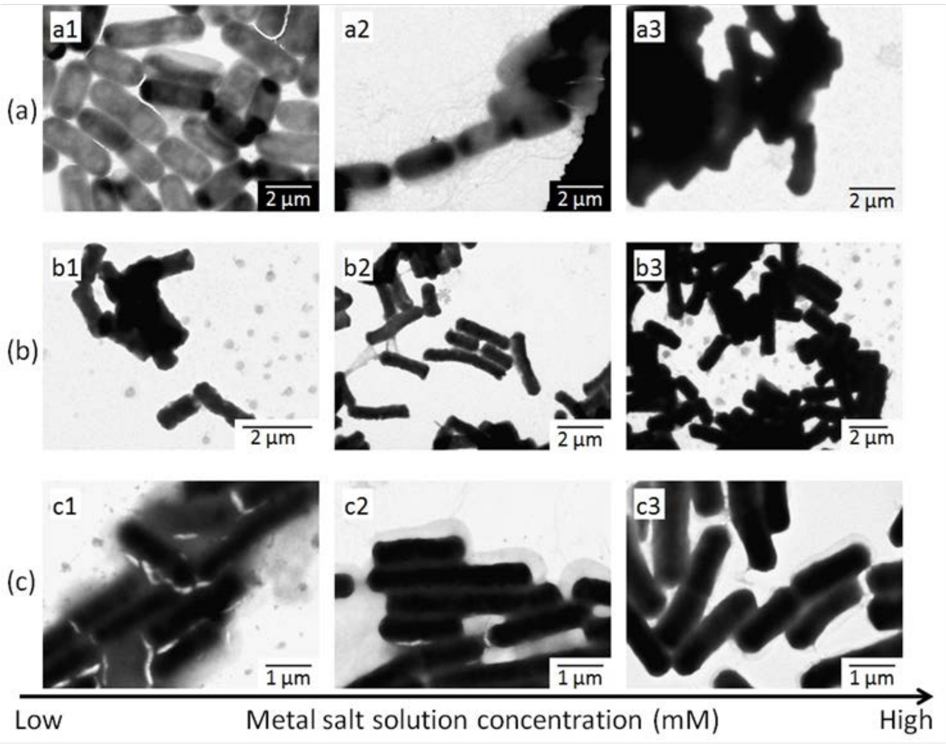

Figure S10. TEM images after adding the metal salt solution which cannot be synthesized into nanoparticles by K-142. (**a**) $AlCl_3$. (**b**) $PtCl_2$. (**c**) $CuCl_2$. Low is 0.1 mM, Middle is 3 mM and High is 5 mM.

TableS1. The conditions of the XRD measurement.

Start Angle	Stop Angle	Sampling Width	
**5.000**	100.000	0.050	
**Scan Speed**	**Voltage**	**Electric Current**	
5.0	40	30	
**Divergence Slit**	**Divergentvertical Slit**	**Scattering Slit**	**Receiving Slit**
1/4°	10 mm	8 mm	Open

Table S2. Names and the sampling locations of the selected strains in the first round of screening.

Names of the Selected Stains	The Sampling Location
K-13, K-19, K-29, K-32	Kamioka-cho wasabo stream
K-49, K-62, K-63, K-86,K-87, K-90	The Takabara creek
K-98, K-108, K-119, K-123	The tributary of Gamata creek (near by the hot spring)
K-140, K-141, K-142, K-149	The tributary of Gamata creek(near by the bus stop)
K-165, K-166, K-167	The Gamata creek
K-237, K-255	The tributary of Takabara creek
K-268	The waterfall nearby trup farm

Table S3. The digitizational results of small amount synthesis of the target strains.

Name of the Stain	Values							Total
	The First Time	The Second Time	The Third Time	The Fourth Time	The Fifth Time	The Sixth Time	The Seventh Time	
K-13	1	0	0	1	1	1	0.5	4.5
K-19	0	0	0	0	0	0	0	0
K-29	0	0	0	0	0.5	0	0	0.5
K-32	0	0	0	0	0	0	0	0
K-49	0	1	1	1	1	0.5	0.5	5.0
K-62	1	1	1	0	0.5	1	0.5	5.0
K-67	0	0	0	0	0	0	0	0
K-86	1	1	1	0.5	0.5	0.5	1	5.5
K-87	0	0	0	0	0	0	0	0
K-90	0.5	0.5	0	0.5	0	0	0	1.5
K-98	1	0	0	0.5	1	1	1	4.5
K-108	1	1	1	1	1	0.5	0.5	6.0
K-119	0	1	1	1	0.5	0	1	4.5
K-123	0	1	1	1	0.5	0.5	1	5.0
K-140	1	0.5	0.5	0.5	0.5	1	0	4.0
K-141	1	0	1	1	1	0.5	1	5.5
K-142	0.5	1	1	1	1	0.5	1	6.0
K-149	0	0	0	0	0	0	0	0
K-165	0	0	0	0	0.5	0	0	0.5
K-166	0	0	0	0	0	0	0	0
K-167	0	0	0	0	0	0	0	0
K-237	0	0	0	0	0	0	0	0

K-255	0	0	0	0	0	0	0	0
K-268	0	0	0	0	0	0	0	0

Table S4. The digitizational results of large amount synthesis of the target strains.

**Name of the Stain**	**Values**			**Total**
	**The First Time**	**The Second Time**	**The Third Time**	
K-13	0	0	0.5	0.5
K-49	1	0.5	0	1.5
K-62	1	0.5	0	1.5
K-86	1	1	0.5	2.5
K-98	0	1	0	1.0
K-108	0.5	1	0.5	2.0
K-119	0.5	0	0	0.5
K-123	1	0.5	0.5	2.0
K-141	0	0	0.5	0.5
K-142	1	1	1	3.0

© 2020 by the authors. Licensee MDPI, Basel, Switzerland. This article is an open access article distributed under the terms and conditions of the Creative Commons Attribution (CC BY) license (http://creativecommons.org/licenses/by/4.0/).
